# Multi-Omics Analysis Reveals the Gut-Mediated Mechanism Underlying the Seasonal Non-Laying Phenotype in Zhedong White Geese (*Anser cygnoides domesticus*)

**DOI:** 10.3390/ani16121899

**Published:** 2026-06-18

**Authors:** Kai Shi, Xiao Zhou, Kai Li, Jiuli Dai, Yangyang Shen, Zhihao Wu, Xinyin Zhang, Quanfa Yu, Shufang Chen

**Affiliations:** 1 Institute of Livestock and Poultry Research, Ningbo Academy of Agricultural Sciences, Ningbo 315000, China; shikai2024@126.com (K.S.); xiaozhou_2023@163.com (X.Z.); xndxlk@139.com (K.L.); 13858357201@163.com (J.D.);; 2 Key Laboratory of Crop-Animal Circular Ecological Agricultural Technology, Ministry of Agriculture and Rural Affairs, Ningbo 315040, China; 3 Ningbo Key Laboratory of White Goose Germplasm Resources Innovation and Ecological Breeding, Ningbo 315000, China; 4 Institute of Animal Science, Jiangsu Academy of Agricultural Sciences, Nanjing 210000, China; sy2azy@163.com

**Keywords:** Zhedong white geese, egg-laying, gut microbiota, metabolome

## Abstract

As a precious indigenous goose resource in China, the Zhedong white goose occupies an essential position in the domestic goose industry. However, this breed spontaneously enters a prolonged non-laying period of over two months per year, which greatly limits egg production capacity and restricts the economic development of the goose industry. Herein, this study systematically compared serum physiological indices and serum and fecal metabolome, as well as fecal microbial communities, between laying and non-laying Zhedong white geese, aiming to reveal the key regulatory mechanisms underlying reproductive stage transition. Physiological analyses indicated that non-laying geese had higher serum levels of GnRH, PRL, APOA, and T-AOC, whereas the concentrations of LH, E_2_, TNF-α, IL-1, and calcium were significantly reduced; FSH, PROG, and BA levels showed no significant differences between the two groups. Metabolomic analysis identified 277 upregulated and 403 downregulated DAMs in feces, and 386 DAMs in serum. The shared enriched pathways across serum and fecal samples encompassed arginine biosynthesis, histidine metabolism, and pantothenate and CoA biosynthesis, as well as steroid hormone biosynthesis. A total of 120 DAMs overlapped in two specimens, and the non-laying geese presented pronounced depletion of tryptophan-derived metabolites and steroid hormone-related metabolites. Metagenomic results showed no significant difference in gut microbial alpha diversity between groups, while their microbial community structures were clearly differentiated. A total of 774 upregulated and 854 downregulated microbial species were screened in non-laying geese, and these differential microbes were primarily enriched in pathways associated with reproductive hormone signaling, steroid biosynthesis and energy metabolism. Multi-omics correlation analysis verified close associations between differential microbes and reproductive-related metabolites. Certain probiotic strains, including *Pediococcus pentosaceus* and *Lactococcus raffinolactis*, were positively correlated with steroid hormones and tryptophan metabolites, and their abundances declined obviously in the non-laying stage. Collectively, this study elaborates the holistic changes in serum biochemistry, gut metabolome and microbiome in geese at different reproductive stages. The dysregulation of amino acid and steroid hormone metabolism, combined with the loss of beneficial intestinal microbes, jointly induces the non-laying phenotype. This study provides new perspectives for understanding the gut–reproductive axis and supplies promising biomarkers to improve the laying performance of geese.

## Simple Summary

The Zhedong white goose is a valuable local breed in China, but its long annual non-laying period hinders industrial profitability. In this work, we compared serum physiological parameters, metabolome and gut microbiota between laying and non-laying geese. Distinct differences in reproductive hormones, inflammatory factors and antioxidant indices were found between the two groups. A large number of differential metabolites were detected in serum and feces, with amino acid and steroid hormone metabolism being the most affected pathways, and key tryptophan and steroid-related metabolites decreased in non-laying geese. Although gut microbial richness and diversity remained stable, community composition varied greatly, and numerous microbial species changed in abundance. Several beneficial bacteria closely linked to reproductive metabolites were markedly reduced during the non-laying stage. Overall, disrupted metabolism and the decline in beneficial gut microbes together lead to the non-laying state. This study uncovers the gut–reproductive regulatory network and offers potential biomarkers to enhance goose egg production.

## 1. Introduction

Geese are economically vital poultry worldwide and play an indispensable role in the agricultural industry, providing high-quality meat, eggs, and valuable by-products such as foie gras and goose down. Benefiting from the high nutritional value of goose-derived products, China has developed a large-scale and mature goose-rearing industry. At present, 15 commercial goose breeds are widely raised across the country, contributing approximately 95% of global goose meat production [1]. According to reproductive photoperiod characteristics, geese can be categorized into three ecotypes: obligate long-day breeders that lay eggs in spring and early summer, partial long-day breeders with reproductive activity from autumn to the subsequent spring, and short-day breeders that lay eggs from late summer to spring [2]. The inevitable seasonal non-laying period markedly reduces annual egg yield; therefore, shortening the non-laying phase is considered a feasible strategy to promote the sustainable development of the goose industry.

Egg-laying is a fundamental reproductive trait for geese to sustain population renewal, and this biological process is tightly modulated by multiple internal and external factors, including photoperiod, hormonal homeostasis, and genetic characteristics. Seasonal fluctuations in photoperiod, ambient temperature, precipitation and humidity act as critical environmental cues that reshape hormone secretion via the hypothalamic–pituitary–ovarian (HPG) axis and ultimately regulate avian reproductive activities [3]. The hypothalamus perceives and integrates external photoperiodic signals to secrete gonadotropin-releasing hormone (GnRH), which further stimulates the pituitary to synthesize and release luteinizing hormone (LH) and follicle-stimulating hormone (FSH). These two core gonadotropins collectively govern ovarian development and laying performance in geese [4]. Additionally, prolactin (PRL) secreted by the anterior pituitary serves as a pivotal regulator of incubation behavior and hypothalamic dopaminergic metabolism in avian species [5]. Previous studies have confirmed that goose laying rates and the developmental status of reproductive organs (ovary and pineal gland) vary dynamically across the four seasons, indicating that photoperiod manipulation can be optimized to improve reproductive efficiency [6]. Further research has verified that the non-laying state is characterized by decreased LH and elevated PRL concentrations; appropriately extending the photoperiod can downregulate PRL secretion while upregulating LH release, thereby reactivating egg-laying behavior [7,8]. To date, artificial photoperiod regulation has become the most efficient and widely applied approach to ameliorate the laying performance of commercial geese [9].

The goose gastrointestinal tract contains an abundance of microbes, and large studies proved that gut microbes could act as endocrine-like organs of the gut–brain axis, gut–liver axis, gut–ovary axis and gut–heart axis to affect host growth [10], reproduction [11] and immunity [12]. Compared with sterile mice, microbially colonized mice showed a higher reproductive hormone level and capacity [13]. Sex-dependent discrepancies in gut microbial composition and metabolic function have also been observed in poultry: male cocks harbor higher abundances of Bacteroides and Megamonas and prefer glycan metabolism, whereas hens are dominated by lipid metabolism in cecal microbiota [14]. For laying hens, cecal microbiota undergoes staged succession throughout the reproductive cycle, with four distinct phases characterized by the proliferation of Proteobacteria, absolute dominance of Firmicutes, competitive replacement of Bacteroidetes by Firmicutes, and a stable Firmicutes/Bacteroidetes ratio [15]. A higher relative abundance of Bacteroidetes is positively correlated with egg quality and laying rate, which is attributed to enhanced nutrient absorption and utilization [16]. Moreover, specific intestinal microbes encode β-glucuronidase (gmGUS), a key enzyme that mediates estrogen enterohepatic recycling and modulates circulating hormone concentrations [17]. A previous goose-related study reported significant shifts in gut microbial composition between laying and non-laying stages; differentially enriched Desulfobacterota, Fusobacteriota and Spirochaetota were identified in non-laying geese, and *Bacteroides fragilis* was verified as a core microbial biomarker capable of modulating serotonin and PRL secretion to affect broodiness [18].

The Zhedong white goose is a premium indigenous meat-type goose breed endemic to eastern China. As a typical short-day breeder, this breed presents strict seasonal reproductive characteristics, with its natural laying period spanning from September to May of the next year, accompanied by intense intrinsic broody tendency [19]. The spontaneous three-month non-laying period causes massive feed waste and severe economic loss of goose farming. In the present study, we systematically compared serum hormonal profiles and biochemical parameters between laying and non-laying Zhedong white geese. Combined fecal metabolomics and metagenomics were further performed to screen differential metabolites and gut microbes. Our findings aim to reveal the potential gut–microbe–metabolite–hormone regulatory mechanism of the non-laying phenotype and provide targeted theoretical references for optimizing laying performance in geese.

## 2. Materials and Methods

### 2.1. Animals

A total of 19 healthy two-year-old female Zhedong white geese (4183 ± 407 g) were selected from a reservoir population of approximately 1000 female geese. All experimental geese were reared under uniform conventional husbandry conditions at a commercial goose farm in Ningbo, Zhejiang Province, China. The geese were housed in indoor pens equipped with outdoor playground areas and had ad libitum access to standard commercial feed and drinking water under natural photoperiod and ambient temperature conditions. Sample collection was performed at two typical reproductive stages corresponding to the biological characteristics of Zhedong white geese: 10 individuals (3888 ± 355 g) were assigned to the laying group and sampled in October (natural laying period), while the remaining nine geese (4508 ± 146 g) constituted the non-laying group and were sampled in July (seasonal non-laying period). To avoid confounding effects caused by body weight differences on physiological and metabolic profiles, geese with comparable body weights were rigorously matched between the two groups before sampling.

Prior to sampling, all geese were subjected to 12 h overnight fasting to minimize the interference of dietary metabolites. Blood samples were aseptically collected from the wing vein of each goose. All blood was naturally coagulated at room temperature and centrifuged at 3000× *g* for 15 min at 4 °C to isolate serum, which was subsequently used for the detection of hormones, biochemical indicators and untargeted LC-MS/MS metabolomic analysis. Meanwhile, fresh fecal samples were directly collected from the cloaca of each live goose during defecation. All collected serum and fecal samples were immediately snap-frozen in liquid nitrogen and stored at −80 °C for subsequent metabolomic profiling and metagenomic sequencing.

### 2.2. Serum Indicator Detection

Appropriate commercial kits (Shanghai Biological Technology Co., Ltd., Shanghai, China) were used to determine serum GnRH (YJ505741), PRL (ML640701V), LH (ML620399V), FSH (YJ64125), PROG (YJ612543), E_2_ (YJ604025), APOA (ml092616), BA (YJ220458), T-AOC (ML035), TNF-α (ml522102V), IL-1 (ml659914) and total calcium (ML036) with an Infinite M200 microplate reader (Tecan Group Ltd., Männedorf, Switzerland) following the manufacturer’s instructions. All measurements were performed with three technological replicates.

### 2.3. Metabolomic Analysis

Metabolites were extracted from goose serum and feces by grinding samples in liquid nitrogen, followed by resuspension in pre-chilled 80% methanol. Quality control (QC) samples were generated by pooling equivalent volumes of metabolites from all individual samples, and blank samples were simultaneously prepared to subtract background ion interference. Untargeted metabolomic profiling was conducted via an LC MS/MS platform under both positive and negative electrospray ionization modes for relative metabolite quantification. Metabolites were preliminarily characterized based on matched retention time and mass-to-charge (*m*/*z*) features against authoritative public databases. Raw metabolomic data were preprocessed by background subtraction using blank samples and unified data normalization. All annotated metabolites were cross-referenced against three mainstream databases, including KEGG (https://www.genome.jp/kegg/pathway.html, accessed 10 February 2026), HMDB (https://hmdb.ca/metabolites, accessed 20 February 2026), and LIPIDMaps (http://www.lipidmaps.org/, accessed 22 February 2026). In this study, metabolites with a variable importance in projection (VIP) > 1, *p*-value < 0.05, and fold change ≥2 or ≤0.5 were defined as differentially accumulated metabolites (DAMs). Functional enrichment analysis of screened DAMs was performed using the online platform MetaboAnalyst (Version 6.0) [20].

### 2.4. Metagenome Analysis

Total genomic DNA was extracted from feces collected from 19 Zhedong white geese, comprising 9 non-laying geese and 10 laying geese. The concentration of extracted DNA was quantified, and DNA integrity was assessed via 1% agarose gel electrophoresis. After rigorous quality control, 0.2 μg of qualified genomic DNA was sheared into fragments with an average length of 400 bp using the Covaris M220 ultrasonication system (Gene Company Limited, Shanghai, China). The fragmented DNA was used to construct paired-end sequencing libraries with the NEXTFLEX Rapid DNA-Seq Kit (Bioo Scientific, Austin, TX, USA), following the manufacturer’s standard protocols. Briefly, sequencing adapters containing complete primer hybridization sites were ligated to the blunt ends of DNA fragments. The prepared libraries were subsequently sequenced on the Illumina NovaSeq platform (Illumina Inc., San Diego, CA, USA) at Majorbio Bio-Pharm Technology Co., Ltd. (Shanghai, China), using the NovaSeq 6000 S4 Reagent Kit v1.5 (300 cycles) according to official guidelines (www.illumina.com). Raw paired-end reads were preprocessed with fastp (v0.20.0) to remove adapter sequences and low-quality reads; filtering criteria included read lengths below 50 bp, quality values lower than Q20, and ambiguous N bases. The clean reads were mapped to the goose reference genome (GCF_964211835.1) using BWA (v0.7.9a), and host-derived reads as well as their paired sequences were discarded. De novo metagenomic assembly was performed with MEGAHIT (v1.1.2) based on succinct de Bruijn graphs to generate assembled contigs [21]. Open reading frames (ORFs) within each contig were predicted using Prodigal. Only ORFs longer than 100 bp were retained and translated into amino acid sequences according to the NCBI genetic code table [22]. A non-redundant (NR) gene catalog was established using CD-HIT (v4.6.1) with a 90% sequence identity threshold and 90% coverage cutoff [23]. Representative genes from the NR catalog were taxonomically annotated against the NCBI NR database using Diamond (v0.8.35) with an e-value of 10^−5^. Meanwhile, Diamond was also used for functional annotation against the eggNOG database for COG classification and the KEGG database for biological pathway enrichment, with an identical e-value threshold.

### 2.5. Statistical Analysis

Each goose was regarded as an individual experimental unit. All statistical analyses were conducted using R software (v4.5.1) and GraphPad Prism (v10.0). Serum data were compared through unpaired Student’s *t*-tests and presented as the mean ± SD. Non-parametric Mann–Whitney U tests and Wilcoxon rank-sum tests were adopted to screen differentially abundant microbial taxa (including phylum, genus, and species) and metabolites. Spearman’s rank correlation analysis was applied to evaluate the correlation between bacterial abundance and metabolite profiles across all samples. The Benjamini–Hochberg false discovery rate (FDR) method was employed to correct *p*-values for multiple testing, and a threshold of *p*-value < 0.05 was considered statistically significant throughout this study.

## 3. Results

### 3.1. Serum Parameter Profiles of Geese During Laying and Non-Laying Stages

**Figure 1:**
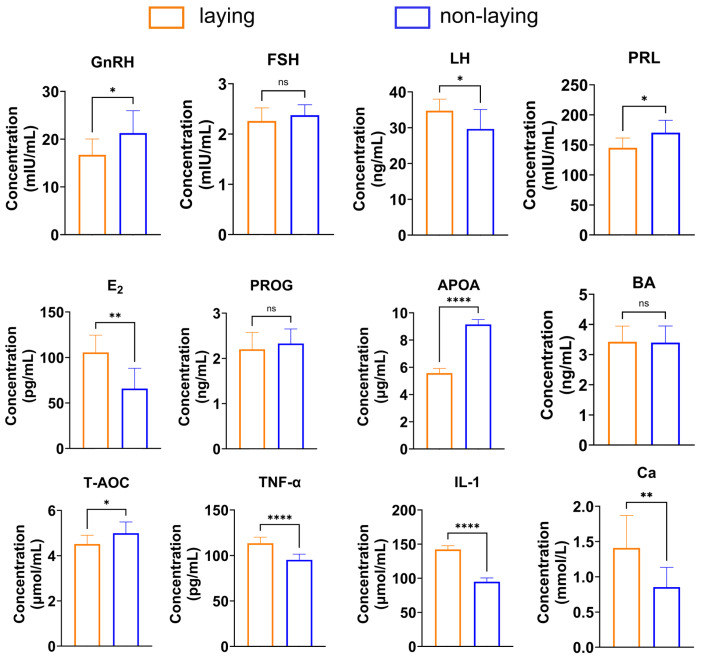
The concentrations of serum parameters in Zhedong white geese during laying and non-laying periods. GnRH: gonadotropin-releasing hormone; FSH: follicle-stimulating hormone; LH: luteinizing hormone; PRL: prolactin; E_2_: estradiol; PROG: progesterone; APOA: apolipoprotein A; BA: bile acid; T-AOC: total antioxidant capacity; TNF-α: tumor necrosis factor-alpha; IL-1: interleukin-1; Ca: calcium. Asterisks indicate statistical significance: * *p*-value < 0.05, ** *p*-value < 0.01, **** *p*-value < 0.0001; ns, not significant.

Compared with the laying period, the serum concentrations of GnRH, PRL, APOA, and T-AOC were significantly elevated in Zhedong white geese during the non-laying period. In contrast, the non-laying geese exhibited markedly lower serum concentrations of LH, E_2_, TNF-α, IL-1, and Ca. No significant differences in FSH, PROG, and BA were detected between the two reproductive stages (Figure 1).

### 3.2. Overview of Serum and Fecal Metabolomic Profiles Across Different Reproductive Stages

**Figure 2:**
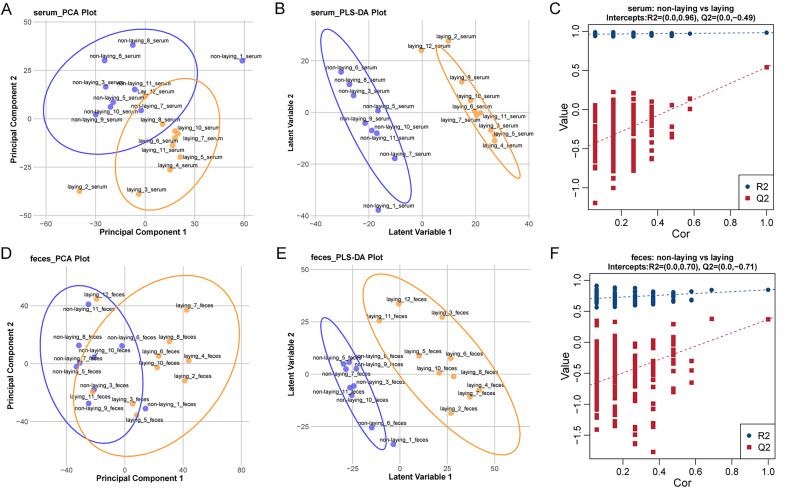
Multivariate statistical analysis of serum and fecal metabolic profiles between laying and non-laying geese. (**A**) PCA score plot of serum metabolites; (**B**) PLS-DA score plot of serum metabolites; (**C**) permutation test of the serum PLS-DA model; (**D**) PCA score plot of fecal metabolites; (**E**) PLS-DA score plot of fecal metabolites; (**F**) permutation test of the fecal PLS-DA model.

LC-MS/MS-based untargeted metabolomics was performed to characterize the metabolic variations between laying and non-laying geese. In total, 1352 metabolites were identified under negative ionization mode, and 2005 metabolites were annotated under positive ionization mode. PCA and PLS-DA analysis indicated a distinct separation of serum (Figure 2A,B) and fecal (Figure 2D,E) metabolic profiles between the laying and non-laying groups. Permutation tests were conducted to assess model reliability and exclude potential overfitting (Figure 2C,F).

**Figure 3:**
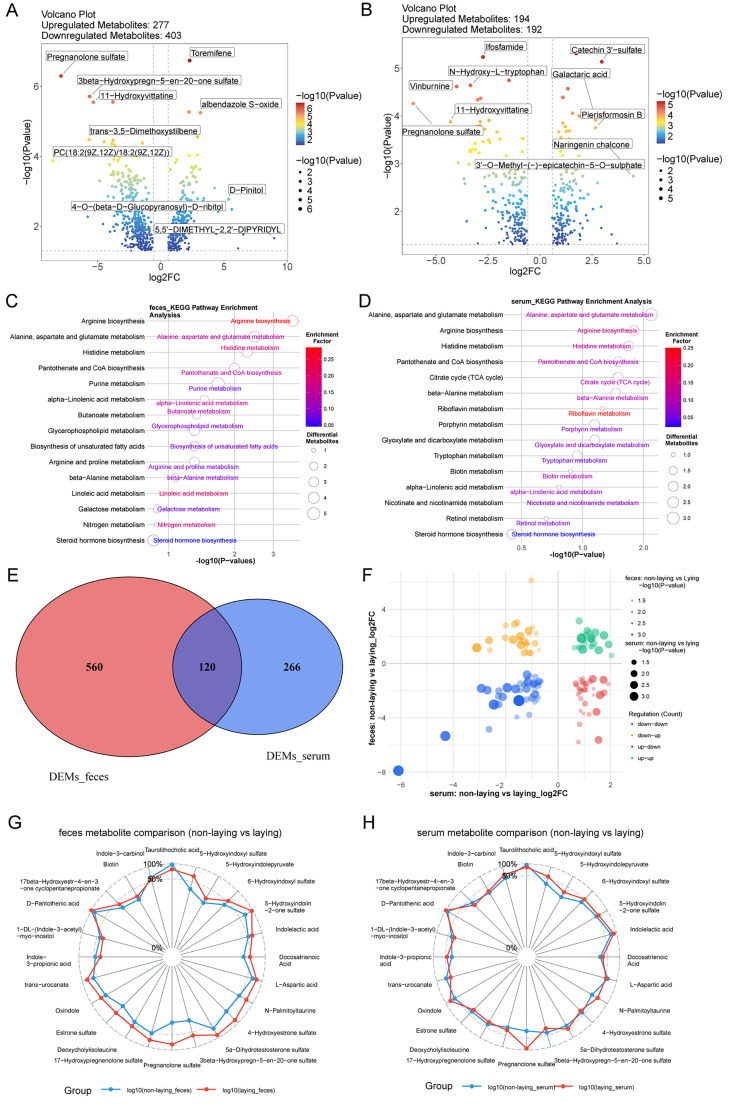
Screening and functional analysis of DAMs between laying and non-laying geese. (**A**) Volcano plot of differential metabolites in feces between non-laying and laying geese; (**B**) volcano plot of differential metabolites in serum from different groups; (**C**) KEGG enrichment analysis of DAMs in feces; (**D**) KEGG enrichment analysis of DAMs in serum; (**E**) Venn diagram of differential metabolites between serum and feces; (**F**) quadrant correlation analysis of shared differential metabolites in serum and feces; (**G**) radar plot of fecal differential metabolite expression patterns; (**H**) radar plot of serum differential metabolite expression patterns.

A total of 277 upregulated differentially accumulated metabolites (DAMs, e.g., toremifene, albendazole S-oxide, and D-pinitol) and 403 downregulated DAMs (e.g., pregnanolone sulfate, 3beta-hydroxypregn-5-en-20-one sulfate, and 11-hydroxyvittatine) were screened in feces of non-laying geese (Figure 3A and Table S1). These DAMs were primarily enriched in arginine biosynthesis, histidine metabolism, and pantothenate and CoA biosynthesis, as well as steroid hormone biosynthesis (Figure 3C). A total of 386 serum DAMs (e.g., pregnanolone sulfate, galactaric acid, and ifosfamide) were identified between non-laying and laying geese (Figure 3B and Table S2). These DAMs were enriched in identical functional pathways to those characterized in fecal samples, including arginine biosynthesis, histidine metabolism, pantothenate and CoA biosynthesis, and steroid hormone biosynthesis (Figure 3D). Subsequent cross-comparison of DAMs between serum and feces showed that 560 DAMs were uniquely present in feces, 266 DAMs were specifically detected in serum, and 120 DAMs were shared across the two sample types (Figure 3E). Among these overlapping metabolites, 39 DAMs were consistently downregulated and 24 DAMs were consistently upregulated in non-laying geese (Figure 3F). Notably, multiple tryptophan-derived metabolites (indolelactic acid, 5-hydroxyindoxyl sulfate, and 5-hydroxyindolepyruvate) and steroid hormone-related metabolites (5α-dihydrotestosterone sulfate, pregnanolone sulfate, and 17-hydroxypregnenolone sulfate) were synchronously depleted in both serum and feces of non-laying geese (Figure 3G,H).

### 3.3. Alterations in Fecal Microbial Composition and Function Revealed by Metagenomic Sequencing

**Figure 4:**
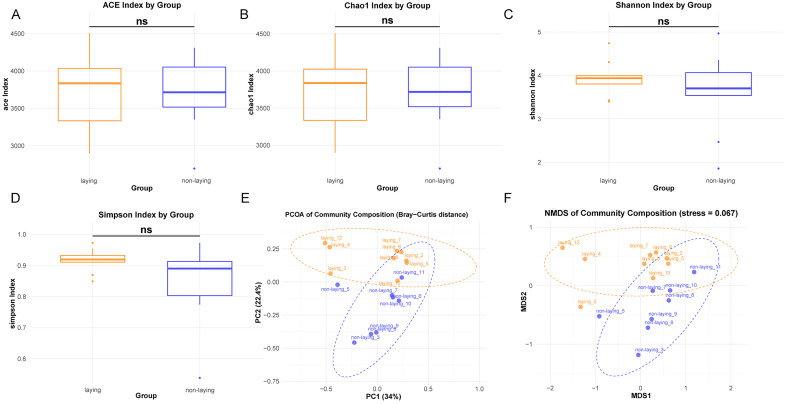
Comparison of fecal microbial alpha and beta diversity between laying and non-laying geese. (**A**) ACE index; (**B**) Chao1 index; (**C**) Shannon index; (**D**) Simpson index. (**E**) Bray–Curtis distance-based PCoA ordination; (**F**) Bray–Curtis distance-based NMDS ordination. ns indicates no significant difference.

Metagenomic sequencing was conducted to explore the differences in microbial community structure and functional potential between laying and non-laying geese. After data filtering and host sequence removal, approximately 1.2 G clean reads were generated, with an average of 34.1 M reads per sample. No significant differences in fecal microbial richness were detected between laying and non-laying geese according to ACE and Chao1 indices (Figure 4A,B). Likewise, Shannon and Simpson alpha diversity showed no inter-group discrepancies in microbial community diversity (Figure 4C,D). However, PCoA and NMDS ordinations demonstrated obvious separation of fecal microbial community structures between the two groups (Figure 4E,F).

**Figure 5:**
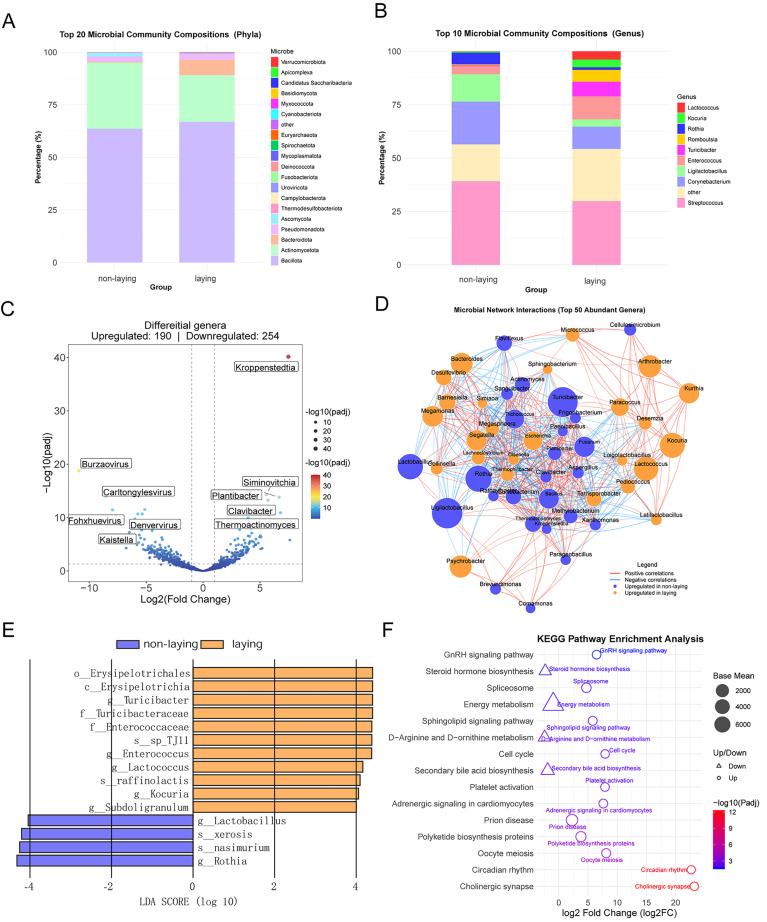
Metagenomic analysis of fecal microbiota from Zhedong white geese during laying and non-laying periods. (**A**,**B**) Taxonomic distribution of fecal microbiota at the phylum and genus levels; (**C**) volcano plot showing differentially abundant genera between the two groups; (**D**) interaction network of differential microbial taxa; (**E**) LEfSe cladograms for comparison of bacterial taxa; (**F**) functional enrichment analysis of differential microbiota.

Taxonomic annotation demonstrated that Bacillota, Actinomycetota, Bacteroidota, Pseudomonadota, and Ascomycota constituted the dominant bacterial phyla across all samples (Figure 5A). At the genus level, *Streptococcus*, *Corynebacterium*, *Ligilactobacillus*, and *Enterococcus* dominated the fecal microbiota of both groups (Figure 5B). Differential analysis showed that 190 genera were enriched and 254 genera were depleted in the non-laying group (Figure 5C). Three core genera, namely *Lactococcus*, *Latilactobacillus*, and *Loigolactobacillus*, were significantly decreased in non-laying geese and presented positive interactive correlations with each other (Figure 5D). LEfSe analysis identified *Turicibacter*, *Enterococcus*, *Lactococcus*, *Kocuria*, and *Subdoligranulum* as microbial biomarkers for the laying group, whereas *Lactobacillus* and *Rothia* served as signature taxa for the non-laying group (Figure 5E). KEGG functional analysis indicated that pathways related to GnRH signaling, oocyte meiosis, circadian rhythm, and cholinergic synapse were upregulated in non-laying geese, while steroid hormone biosynthesis, secondary bile acid biosynthesis, energy metabolism, and D-arginine/D-ornithine metabolism were significantly suppressed (Figure 5F).

### 3.4. Integrative Analysis of Fecal Microbiome, Metabolome and Serum Parameters

**Figure 6:**
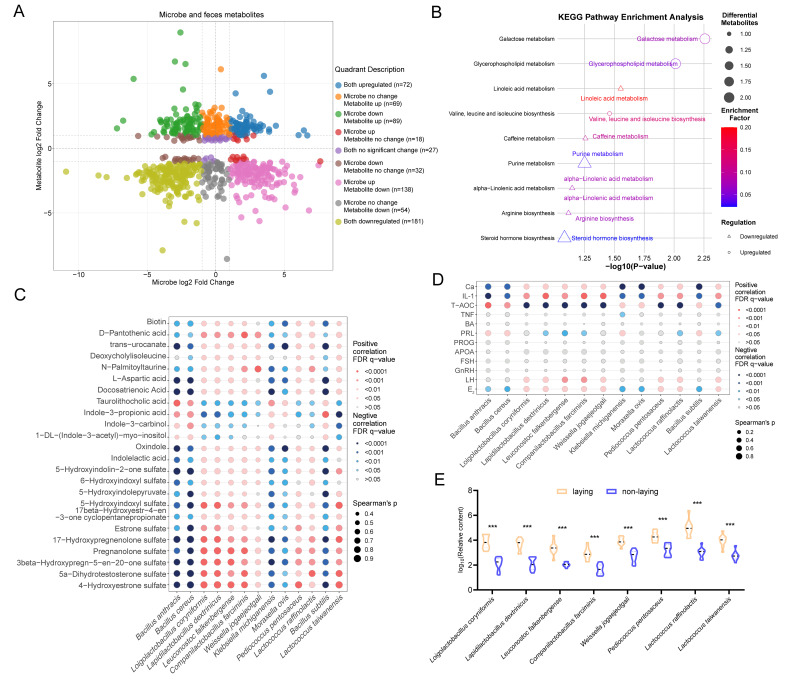
Conjoint analysis of fecal microbiome and metabolome. (**A**) Nine-quadrant plot of differential metabolites and microbiota in feces from laying and non-laying geese; (**B**) KEGG pathway analysis of upregulated and downregulated fecal metabolites; (**C**) bubble plot showing the correlation between differential metabolites and microbiota; (**D**) the correlation analysis between serum parameters and fecal microbiota; (**E**) relative abundance of key fecal microbial species. *** indicates the *p*-value < 0.001.

A nine-quadrant joint analysis was performed to elucidate the intrinsic correlation between fecal microbes and differential metabolites (Figure 6A). A total of 72 upregulated and 181 downregulated microbe–metabolite pairs exhibited consistent variation trends. KEGG enrichment showed that upregulated metabolites were mainly involved in galactose metabolism; glycerophospholipid metabolism; and valine, leucine and isoleucine biosynthesis. By contrast, downregulated metabolites were predominantly associated with steroid hormone biosynthesis, linoleic acid metabolism, arginine biosynthesis, and purine metabolism (Figure 6B). Correlation analysis confirmed that six microbial species, including *Pediococcus pentosaceus*, *Lactococcus raffinolactis*, and *Lactococcus taiwanensis*, were positively correlated with the abundances of estrone sulfate, pregnanolone sulfate, and indolelactic acid (Figure 6C). In addition, the relative contents of *Pediococcus pentosaceus* and *Lactococcus raffinolactis* were positively related to the concentrations of IL-1, E_2_ and LH (Figure 6D). Importantly, these beneficial microbial species were markedly decreased during the non-laying period (Figure 6E).

## 4. Discussion

China dominates global goose farming, contributing 95% to 97% of the world’s total production, and recorded over 736 million slaughtered geese in 2024 [24]. As one of the most valuable indigenous goose breeds in China, the Zhedong white goose plays an indispensable role in supporting the sustainable development of the domestic goose industry [25]. Nevertheless, this breed is characterized by a lengthy non-laying period of more than two months, which severely disturbs regular egg-laying cycles and ultimately results in substantial economic losses. Currently, the molecular mechanisms responsible for prolonged non-laying status are poorly understood. Accordingly, the present study integrated metabolomics and metagenomics to compare hormonal and metabolic discrepancies between laying and non-laying geese, aiming to screen and characterize the core regulatory factors governing reproductive performance.

As core regulators of animal reproduction, hormones play a critical role in the reproductive axis. Specifically, GnRH secreted by the hypothalamus acts on the pituitary gland to stimulate the synthesis of FSH and LH; these two hormones travel through the bloodstream to the ovary, where they promote E_2_ production and ultimately drive egg-laying. Our serum analysis showed that non-laying geese had significantly higher concentrations of GnRH and PRL, alongside lower E_2_ concentrations. Reproductive activities are governed by negative feedback regulation; the decline in E_2_ is probably the main cause of elevated GnRH. PRL is an essential hormone modulating egg-laying performance in birds. Previous studies have demonstrated that prolonged photoperiods inhibit PRL secretion and boost LH release, thereby restoring egg production [8]. PRL can also directly target the pituitary to inhibit the secretion of FSH and LH, and further hinders ovarian and follicular development, triggers ovarian atrophy and terminates egg-laying, and eventually induces broodiness [26]. In the present study, serum PRL concentrations were markedly higher in non-laying geese than in laying individuals, which may contribute to the cessation of egg production. In addition, numerous studies have confirmed that antioxidant status, immune function, lipid metabolism and bile acid metabolism are closely associated with egg-laying traits. For this reason, we further determined serum concentrations of APOA, BA, T-AOC, TNF-α, IL-1 and calcium. The results revealed that non-laying geese presented higher APOA and T-AOC contents, while their contents of TNF-α, IL-1 and calcium were significantly reduced. Collectively, the comprehensive changes in these serum parameters lead to the non-laying state of Zhedong white geese.

Metabolomic profiling detected a total of 680 and 386 differentially accumulated metabolites (DAMs) in the serum and feces of laying and non-laying geese, respectively. Functional enrichment analysis indicated that four overlapping metabolic pathways were significantly enriched across both serum and fecal samples, including arginine biosynthesis, histidine metabolism, alpha-linolenic acid metabolism, and steroid hormone biosynthesis. Previous studies have demonstrated that arginine, histidine, and alpha-linolenic acid are critical nutrients for sustaining normal egg-laying performance; insufficient intake of these substances decreases laying rates and facilitates the initiation of broodiness in geese [27,28,29]. Further screening identified 120 shared DAMs between serum and feces. Notably, non-laying geese exhibited pronounced downregulation of two pivotal types of metabolites, namely tryptophan-derived metabolites (indolelactic acid, 5-hydroxyindoxyl sulfate, and 5-hydroxyindolepyruvate) and steroid hormone-related metabolites (5α-dihydrotestosterone sulfate, pregnanolone sulfate, and 17-hydroxypregnenolone sulfate). As an indispensable amino acid for poultry, dietary tryptophan supplementation has been verified to elevate daily egg production and egg mass [30]. Beyond regulating reproductive performance, tryptophan also maintains intestinal epithelial barrier integrity and alleviates intestinal inflammation. It can upregulate indoleacetic acid levels, and tryptophan together with indoleacetic acid synergistically inhibit NLRP3 inflammasome activation, thereby reducing photoperiod-induced intestinal inflammatory injury [31]. In line with our findings, multiple clinical and animal studies have validated that dynamic changes in pregnanolone sulfate, 5α-dihydrotestosterone sulfate, and 17-hydroxypregnenolone sulfate are tightly correlated with ovarian physiological status and reproductive capacity in females [31,32,33]. Taken together, dysregulation in amino acid and steroid hormone metabolism could potentially disrupt intestinal barrier homeostasis and hormonal balance, and may jointly modulate reproductive performance and broodiness in geese.

Due to the distinct metabolic profiles observed in fecal samples between laying and non-laying geese, metagenomic sequencing was further performed to characterize their gut microbial communities. Differential analysis identified substantial differences in microbial composition, yielding a total of 1628 differential microbial species. Among them, the relative abundances of Lactococcus (including *L. raffinolactis* and *L. taiwanensis*) and Pediococcus (primarily *P. pentosaceus*) were markedly reduced in non-laying geese. As a multifunctional probiotic, *L. raffinolactis* participates in the modulation of intestinal steroid metabolism and synthesizes plasmid-encoded circular bacteriocin raffinocyclicin, which efficiently inhibits common avian pathogens such as *Clostridium perfringens* [34,35]. For *P. pentosaceus*, previous studies have confirmed that this strain possesses protease, lipase, and amylase activities without carrying virulence genes; it can optimize blood biochemical parameters and exert anti-inflammatory effects in animal models [36]. Additionally, dietary supplementation with Pediococcus acidilactici, a homologous strain of *P. pentosaceus*, improved sperm quality, testicular function and antioxidant capacity in roosters [37]. Consistently, experiments in zebrafish further validated that *P. acidilactici* could elevate the expression of the reproductive key gene Cyp19a and ultimately enhance animal fertility [38]. Apart from Pediococcus, clinical evidence also supported the reproductive regulatory function of Lactococcus: the decreased abundance of intestinal *L. raffinolactis* was tightly associated with reduced estrogen contents in patients with uterine fibroids [39]. Therefore, fecal Lactococcus and Pediococcus can serve as potential microbial biomarkers for geese reproduction.

Integrated analysis of the fecal metabolome and metagenome revealed 72 upregulated and 181 downregulated microbes and metabolites. KEGG enrichment analysis of these DAMs demonstrated that several metabolic pathways, including steroid hormone biosynthesis, purine metabolism, and arginine biosynthesis, were significantly downregulated, whereas galactose metabolism and glycerophospholipid metabolism were upregulated. Correlation analysis further confirmed that *Lactococcus raffinolactis* and *Pediococcus pentosaceus* were strongly correlated with multiple hormone-associated metabolites as well as serum parameters (including E_2_, LH and IL-1). It is well acknowledged that gut microbiota-derived β-glucuronidase (gmGUS) regulates circulating estrogen concentrations by mediating enterohepatic circulation [40]. However, the genomic evidence confirmed that neither *L. raffinolactis* nor *P. pentosaceus* encodes the gmGUS gene. Therefore, we hypothesize that the decline in the abundance of these two species could act as potential probiotics, maintaining intestinal immune homeostasis and systemic hormonal balance, and thereby benefiting goose laying performance.

Collectively, we investigated serum physiological indicators, fecal metagenome, and serum and fecal metabolomes in laying and non-laying Zhedong white geese, providing new insights into the reproductive performance of this breed. Since metabolomic and metagenomic analyses involve a large number of metabolites and microbial species, the modest sample size may potentially lower the credibility of the results. Meanwhile, variations in ambient temperature, humidity and photoperiod between groups might interfere with serum and fecal compositions. To mitigate these drawbacks, follow-up experiments will integrate artificial reproductive regulation, increased sample size and unified sampling procedures to further validate the present findings.

## 5. Conclusions

In summary, distinct differences in serum biochemistry, metabolome and gut microbiota exist between laying and non-laying Zhedong white geese. Metabolic disorders in amino acid and steroid hormone pathways, together with shifts in gut microbial composition, are tightly associated with the seasonal non-laying trait. Several beneficial bacterial species correlated with reproductive metabolites were significantly reduced in non-laying geese. This work demonstrates that the gut–microbe–metabolite network acts as a critical regulator of goose reproductive performance. The present results deepen our understanding of the gut–reproductive axis and offer practical clues for optimizing laying performance and promoting the sustainable development of the goose industry.

## Data Availability

The sequencing data used in this study are available at the China National Center for Bioinformation and under Genome Sequence Archive (GSA) CRA032653 (metagenome data). The metabolomic sequencing data used in this study are available from https://doi.org/10.6084/m9.figshare.32448417.

## Supplementary Materials

The following supporting information can be downloaded at: https://www.mdpi.com/article/10.3390/ani16121899/s1, Table S1: Differential metabolites in feces between non-laying and laying geese; Table S2: Differential metabolites in serum between non-laying and laying geese.
